# Lymphoid blast crisis after prolonged treatment‐free remission in chronic myeloid leukaemia after tyrosine kinase inhibitor de‐escalation during the COVID‐19 pandemic

**DOI:** 10.1002/jha2.302

**Published:** 2021-11-19

**Authors:** Daniele Avenoso, Dragana Milojkovic, James Clark, Christopher Pocock, Victoria Potter, Deborah Yallop, Guy Hannah

**Affiliations:** ^1^ Department of Haematological Medicine King's College Hospital NHS Foundation Trust London UK; ^2^ Centre for Haematology Imperial College NHS Trust, Hammersmith Hospital London UK; ^3^ East Kent Hospital University NHS Foundation Trust London UK

**Keywords:** CML, COVID‐19, sudden blast crisis, TRF

## Abstract

During the COVID‐19 pandemic, access to health services has been considerably restricted and furthermore, patients have been reluctant to attend for routine monitoring, and this may have had a negative impact in the management of patients affected with haematological disorders. Sudden blast crisis in chronic myeloid leukaemia is categorized as a rapid onset of blastic phase, after a documented ‘optimal’ response to tyrosine kinase inhibitor (TKI) therapy and within 3 months of a normal complete blood count. Herein, we describe a case of patient who developed sudden blast crisis after TKI while in treatment‐free remission.

This case highlights the need for active and stringent surveillance of chronic myeloid leukaemia (CML) patients following tyrosine kinase inhibitor (TKI) discontinuation, to identify the loss of major molecular response (MMR) and prevent progression to advanced phase disease. It is fundamental for patients to avoid missing scheduled BCR‐ABL RT‐qPCR monitoring, and requires engagement not only with the patient, but also within the treating centre and the molecular laboratory. Engaging in treatment‐free remission (TFR) approaches with the requirements of increased molecular monitoring during the SARS‐CoV‐2 pandemic and associated lockdown measures needs to be carefully considered.

TFR is the next frontier in CML management following the induction of deep molecular responses (DMRs) on TKI therapy. TFR can be an ideal target when offering TKI treatment to newly diagnosed patients and influences the choice of first‐line therapy. The feasibility of stopping imatinib was evaluated in the STIM prospective study, which concluded that nearly half of patients were able to maintain DMR off treatment, suggesting a potential curative role of imatinib [1]. Relatively few patients treated with imatinib achieve a sustained DMR necessary to attempt TKI discontinuation. More potent second‐generation TKIs demonstrate higher and earlier rates of the required molecular responses compared with imatinib, offering the opportunity of TFR to a greater number of patients [2, 3].

The condition sine qua non for a safe discontinuation of TKI therapy is the presence of at least 5 years of treatment and 2 years of MR4. Frequent molecular monitoring after stopping therapy is usually recommended monthly for at least the first 6–12 months, then every 2 months for 6–12 months and every 3–6 months thereafter. More than 80% of molecular recurrences occur within the first 6–8 months after stopping TKI. For the 50% of patients who lose MMR after discontinuation, disease control is usually regained upon restarting TKI [4].

The UK‐based DESTINY trial explored the feasibility and safety of TKI dose reduction (imatinib, nilotinib and dasatinib) and then treatment cessation in patients in both stable MMR and DMR. The molecular recurrence‐free survival rate (defined as maintenance of MMR) was found to be 72% in the DMR group and substantially better than that observed in the EUROSKI trial, which had a recurrence‐free survival rate of 50% at 2 years. No progressions to advanced phase occurred within the DESTINY study [5].

**FIGURE 1:**
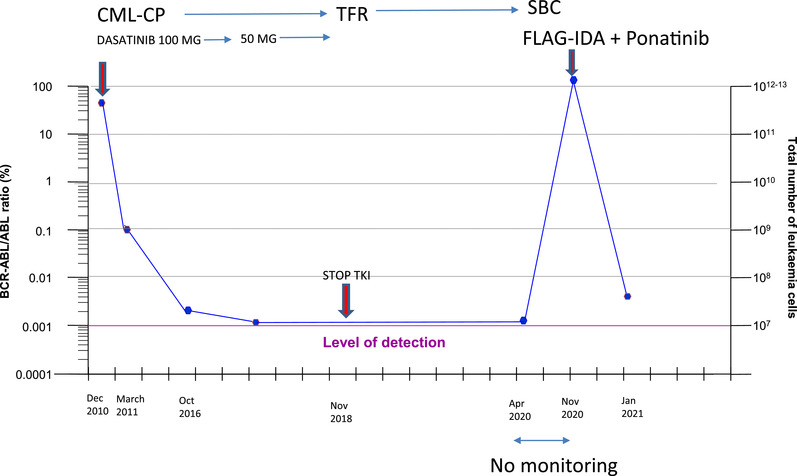
Patientcase summary and molecular monitoring. Abbreviations: CML‐CP, chronic myeloid leukaemia, chronic phase; SBC, sudden blast crisis; TFR, treatment‐free remission; TKI, tyrosine‐kinase inhibitor; FLAG‐IDA, cytarabine 1000 mg/m^2^ from day 1 to 5, fludarabine 30 mg/m^2^ from day 1 to 5, idarubicin 8 mg/m^2^ days 3–5. Dasatinib was de‐escalated to 50 mg for 12 months before stopping it

During the COVID‐19 pandemic, access to health services has been considerably restricted and furthermore, patients have been reluctant to attend for routine monitoring [6]. As molecular monitoring after stopping TKI is an essential component of the safety of this approach, many physicians and patients elected not to commence TFR programmes during this time.

Blast crisis (BC) is well defined, but a more rapid progression to advanced phase has also been recognized. Sudden blast crisis (SBC) is categorized as a rapid onset of BC, after a documented ‘optimal’ response to TK therapy and within 3 months of a normal complete blood count [7]. SBC is a rare event reported during imatinib therapy or after allogeneic stem cell transplant [8]. It has also been previously described after imatinib discontinuation despite re‐achievement of MMR, and the pathogenesis in this setting remains unclear [9]. We describe a case of SBC in a patient who discontinued dasatinib for 24 months within a TFR programme, after prior dasatinib dose‐reduction.

A 46‐year‐old‐man was diagnosed with CML, intermediate Sokal score, in December 2010; he was started on Dasatinib 100 mg in January 2011, obtaining MMR at 4 months, MR4 at 15 months and MR4.5 in October 2016 Figure 1. Prior to TKI discontinuation, a de‐escalation approach, as outlined in the DESTINY trial, was implemented, and after 1 year of Dasatinib 50 mg, he stopped TKI therapy in November 2018. He remained in at least MR4 after stopping dasatinib, but only attended for molecular monitoring for 18 months after TKI discontinuation. Last BCR‐ABL RT‐qPCR analysis was in April 2020, where ongoing CMR5 (0.000% IS) was documented. Unfortunately, he did not attend for either BCR‐ABL or FBC monitoring between April and November 2020 during the COVID‐19 pandemic. In November 2020, the patient developed fatigue, night sweats, anaemia and thrombocytopenia. Bone marrow biopsy showed an infiltrate with B‐cell progenitors (immunophenotype: CD19^+^, CD34^–^, CD10^–^, TdT^+^, CD20^–^, CD38 weak, CD66^+^, MPO^–^, CD33^–^) confirming lymphoid BC, 7 months after CMR was last documented. In addition to the Philadelphia (Ph) chromosome, G‐banded karyotyping showed additional, minor and major route additional chromosome abnormalities: additional Ph chromosome, loss of Y, additional material of unknown origin on the short arm of chromosome 8, an additional copy of chromosome 8 as dicentric isochromosome of the long arm at break point p11; RT‐qPCR BCR‐ABL was 85% IS (e13a2 transcript). He started treatment with FLAG‐IDA and Ponatinib 30 mg [10] achieving second CP, and MR4.5.

To the best of our knowledge, this is the first report of BC in the United Kingdom during a TFR strategy and the fifth world‐wide case of BC post‐TKI discontinuation. Although SBC may have occurred in this case, the last documentation of CMR or complete haematological response was 7 months prior to the presentation with BC.

Immune surveillance is understood to be a key factor in maintaining a durable TFR, and cytotoxic NK cells thought to play a critical role in the suppressive effect on leukaemic stem cells [11]. What is not known, and what the rare cases of SBC while in apparent successful TFR do not yet show, is why immune surveillance can be so dramatically lost. It is important to emphasize that despite the aggressiveness of BC, often thought of as terminal event, SBC in the context of TFR is still highly sensitive to treatment [12]. This suggests that SBC may be a result of the expression of a different clone selected by TKI exposure and immune surveillance and with a potential different prognosis. The rarity of the event limits conclusions regarding long‐term prognosis and despite the successful treatment in the few SBC reported.

This case highlights the need for active and stringent surveillance of CML patients following TKI discontinuation, to identify loss of MMR and prevent progression to advanced phase disease. It is fundamental for patients to avoid missing scheduled BCR‐ABL RT‐qPCR monitoring, and requires engagement not only with the patient, but also within the treating centre and the molecular laboratory. Engaging in TFR approaches with the requirements of increased molecular monitoring during the SARS‐CoV‐2 pandemic and associated lockdown measures needs to be carefully considered.

## AUTHORS CONTRIBUTIONS

DA, GH, DY, VP, JC, CP and DM were involved in the care of the patient, writing and reviewing the manuscript.
